# Modulation of VEGF-A Alternative Splicing as a Novel Treatment in Chronic Kidney Disease

**DOI:** 10.3390/genes9020098

**Published:** 2018-02-15

**Authors:** Megan Stevens, Sebastian Oltean

**Affiliations:** Institute of Biomedical and Clinical Sciences, University of Exeter Medical School, Exeter, EX1 2LU, UK

**Keywords:** VEGF-A, alternative splicing, kidney disease

## Abstract

Vascular endothelial growth factor A (VEGF-A) is a prominent pro-angiogenic and pro-permeability factor in the kidney. Alternative splicing of the terminal exon of VEGF-A through the use of an alternative 3′ splice site gives rise to a functionally different family of isoforms, termed VEGF-A_xxx_b, known to have anti-angiogenic and anti-permeability properties. Dysregulation of the VEGF-A_xxx_/VEGF-A_xxx_b isoform balance has recently been reported in several kidney pathologies, including diabetic nephropathy (DN) and Denys–Drash syndrome. Using mouse models of kidney disease where the VEGF-A isoform balance is disrupted, several reports have shown that VEGF-A_165_b treatment/over-expression in the kidney is therapeutically beneficial. Furthermore, inhibition of certain splice factor kinases involved in the regulation of VEGF-A terminal exon splicing has provided some mechanistic insight into how VEGF-A splicing could be regulated in the kidney. This review highlights the importance of further investigation into the novel area of VEGF-A splicing in chronic kidney disease pathogenesis and how future studies may allow for the development of splicing-modifying therapeutic drugs.

## 1. Introduction

The human genome is comprised of approximately 20,000 genes, but the human proteome is estimated to be formed of hundreds of thousands of proteins [1]. This diversity is mainly the result of a process known as alternative splicing (AS); a single gene transcript can give rise to multiple proteins depending on the way the gene is spliced [2]. In humans, more than 94% of genes can be alternatively spliced [3,4]. It is generally accepted that the more evolved a species is, the higher the percentage of genes that undergo AS [5].

It is becoming increasingly clear that changes to the tightly regulated process of AS in many genes can result in cellular dysfunction and disease. Cancer is the disease that is most commonly linked to AS dysregulation [6,7,8]; however, there are an increasing number of splice isoforms that have been recently implicated in kidney disease, including vascular endothelial growth factor A (VEGF-A) [9,10]. VEGF-A is key driver of angiogenesis, permeability, migration, and cell survival [11]. The abnormal expression of VEGF-A in the kidney has been widely reported in most types of kidney disease [12]. Alternative splicing of exon 8 of the VEGF-A gene results in the expression of an anti-angiogenic, anti-permeability, and cyto-protective family of isoforms, termed the VEGF-A_xxx_b family (the most common isoform being VEGF-A_165_b) [13], which has been shown to be protective in kidney disease [9,10].

This review discusses the mechanism of VEGF-A splicing, highlights the importance of a balance of the VEGF-A exon 8 splice variants in the kidney, and examines the ways in which VEGF-A splicing can be manipulated to obtain therapeutic benefit.

## 2. Alternative Splicing of VEGF-A

### 2.1. Modes of Alternative Splicing

The process of pre-mRNA AS involves the inclusion/exclusion of whole exons or parts of exons/introns in the final mRNA transcript. This process is achieved with a splicing reaction conducted by the spliceosome, a macromolecular assembly composed of small nuclear ribonucleoproteins and the associated accessory proteins [14]. In brief, the spliceosome assembles at a splice site (a conserved sequence in the pre-mRNA transcript at the exon-intron junction) and undergoes two transesterification reactions to achieve splicing. The main modes of AS are: cassette exon skipping or retention, intron retention, use of an alternative 3′ or 5′ splice sites in exons, and the inclusion/exclusion of two mutually exclusive exons.

### 2.2. Regulation of Alternative Splicing

AS is regulated by Cis-acting regulatory elements (auxiliary sequences), which can be divided into four subgroups; exonic and intronic sequence enhancers (ESEs and ISEs), and exonic and intronic sequence silencers (ESSs and ISSs). These Cis-acting regulatory elements recruit trans-acting splice factors to modulate the splicing reaction [2]. Splice factors are RNA-binding proteins, the most common being serine/arginine-rich (SR) proteins, that act with small nuclear ribonucleoproteins to form and mediate the action of the spliceosome [15]. Another key family of splice factors are the heterogeneous ribonucleoproteins (hnRNPs). In general, SR proteins bind to ESEs and recruit core splice factors to the polypyrimidine tract, activating splicing of a cassette exon, whereas hnRNPs classically block access of the spliceosome to the polypyrimidine tract, suppressing RNA splicing [16,17]. However, there are exceptions to these general rules.

### 2.3. Alternative Splicing of VEGF-A

**Figure 1:**
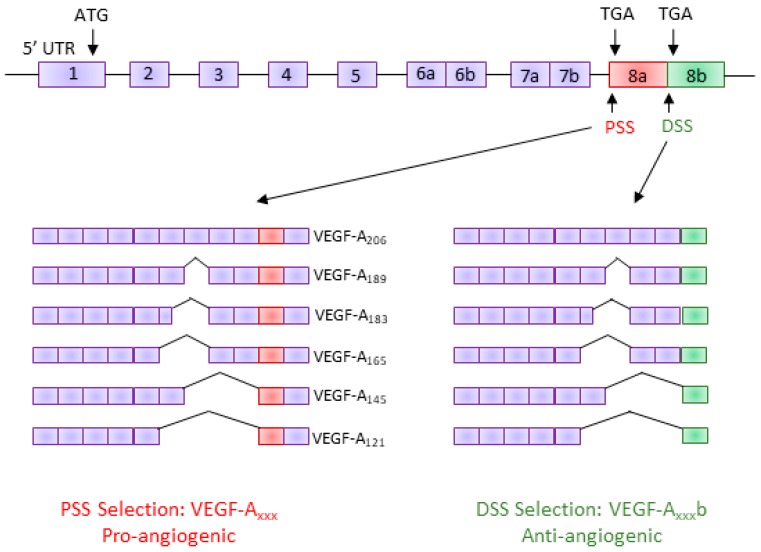
**Vascular endothelial growth factor A (VEGF-A) splice variants.** The VEGF-A pre-mRNA is comprised of eight exons; inclusion/exclusion of these exons gives rise to several isoforms with differing amino acid lengths. In the terminal exon (exon 8), an alternative 3′ splice site results in a new family of isoforms, the VEGF-A_xxx_b family. These isoforms have the same number of amino acids but a different C-terminus sequence, resulting in them being functionally different (anti-angiogenic). PSS: proximal splice site; DSS: distal splice site; UTR: untranslated region.

Human VEGF-A is a major regulator of angiogenesis and vessel permeability [11]. The *VEGF-A* gene consists of eight exons and seven introns [18]. AS of VEGF-A can occur through the inclusion/exclusion of various exons, giving rise to a family of pro-angiogenic isoforms generically known as VEGF-A_xxx_ (VEGF-A_121_, VEGF-A_145_, VEGF-A_165_, VEGF-A_189_, and VEGF-A_206_—the number denotes the number of amino acids) [19]. It was not until 2002 that a novel alternative 3′ splice site (distal splice site; DSS) was discovered in exon 8 of the human VEGF-A gene, 66 base pairs downstream of the canonical proximal 3′ splice site (PSS); use of the DSS creates a new open reading frame and results in a functionally different family of isoforms, termed the VEGF-A_xxx_b family [13]. Although the VEGF-A_xxx_b isoforms have the same number of amino acids in total, they have an altered C-terminal sequence that differs by six amino acids (Figure 1). This small change in the terminal six amino acids results in VEGF-A_xxx_b having functionally opposite properties to VEGF-A_xxx_; VEGF-A_xxx_b has anti-angiogenic, anti-permeability, and anti-migratory properties [9,10,13,20]. However, like VEGF-A_xxx_, VEGF-A_xxx_b is a pro-survival factor [21].

The most common VEGF-A_xxx_b isoform in the kidney is VEGF-A_165_b [22]. The differing properties of the anti-angiogenic VEGF-A_165_b are thought to be due to its inability to efficiently autophosphorylate VEGF receptor 2 (VEGFR-2), the key VEGF-A receptor for driving angiogenesis, permeability, and migration [20]. Several studies have shown that VEGF-A_165_b poorly activates the VEGFR-2 kinase domain, resulting in weak, transient phosphorylation of downstream targets including Akt and ERK1/2 [10,23].

### 2.4. Regulation of VEGF-A Exon 8 Splicing

AS of exon 8 of the VEGF-A gene is known to be regulated by several SR proteins. Pre-mRNA sequence analysis revealed a predicted binding site for Serine/Arginine Rich Splicing Factor 1 (SRSF1) upstream of the DSS, and a predicted binding site for SRSF6 downstream of the DSS [24]. Binding of SRSF1 to the pre-mRNA promotes PSS selection, thus resulting in the expression of the VEGF-A_xxx_ isoforms. On the other hand, binding of SRSF6 to the pre-mRNA promotes DSS selection and the expression of VEGF-A_xxx_b isoforms [25].

**Figure 2:**
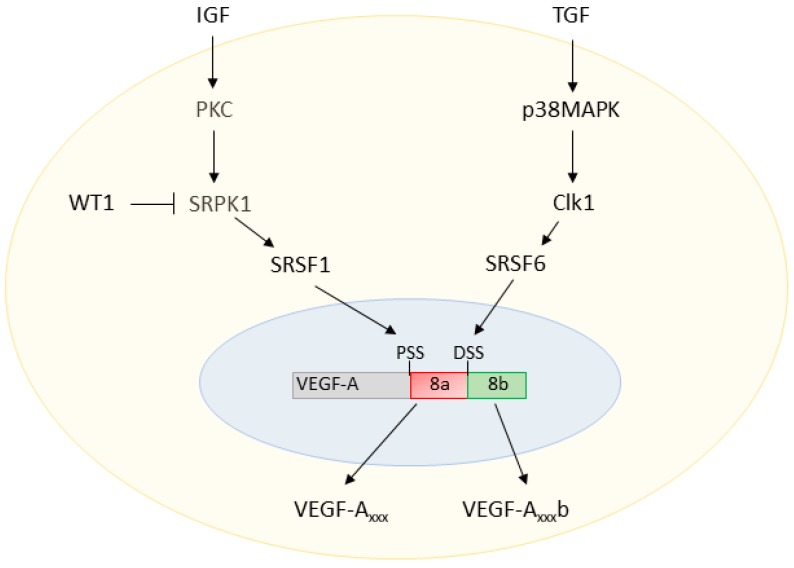
**VEGF-A splicing regulation.** Insulin-like growth factor (IGF) activates protein kinase C (PKC), which in turn phosphorylates SR protein kinase 1 (SRPK1). Activated SRPK1 can then shuttle the splice factor Serine/Arginine Rich Splicing Factor 1 (SRSF1) to the nucleus, resulting in proximal splice site (PSS) selection in exon 8 of VEGF-A. The transcription factor Wilms Tumor 1 (*WT1*) inhibits the synthesis of SRPK1, downregulating PSS selection. On the other hand, transforming growth factor (TGF) signalling activates p38 mitogen-activated protein kinases (p38MAPK), which then activate cell division cycle (CDC)-like kinase 1 (Clk1). Activates Clk1 causes shuttling of the splice factor SRSF6 to the nucleus, resulting in distal splice site (DSS) selection.

The actions of SRSF1 and SRSF6 on the VEGF-A pre-mRNA are modulated by upstream regulators, as summarised in Figure 2. SRSF1 is a known target of the SR protein kinases 1 and 2 (SRPK1/2). Inhibition of SRPK1 activity with small molecule inhibitors or RNA interference (RNAi) knockdown of SRPK1 expression has been reported to block the phosphorylation and nuclear shuttling of SRSF1, switching the ratio of VEGF-A splicing to decrease the pro-angiogenic VEGF-A_xxx_ and increase the anti-angiogenic VEGF-A_xxx_b isoforms [26,27].

AS of the VEGF-A terminal exon is understood to be modified by extracellular factors via signalling cascades, including various growth factors. Insulin-like growth factor 1 (IGF1) and tumour necrosis factor α (TNFα) have been shown to favour the use of the exon 8 PSS through protein kinase C (PKC)-induced activation of SRPK1; however, transforming growth factor β (TGFβ) promotes DSS use through cell division cycle (CDC)-like kinase 1 (Clk1) activation of p38 mitogen-activated kinases (p38MAPK), which phosphorylates SRSF6 [20,25].

Recent work has focused on small molecule inhibitors of SRPK1, such as SRPIN340, to switch the splicing of VEGF-A exon 8 to increase the VEGF-A_165_b/VEGF-A_165_ ratio for therapeutic use [28,29].

## 3. VEGF-A Splice Variants in Chronic Kidney Disease

**Figure 3:**
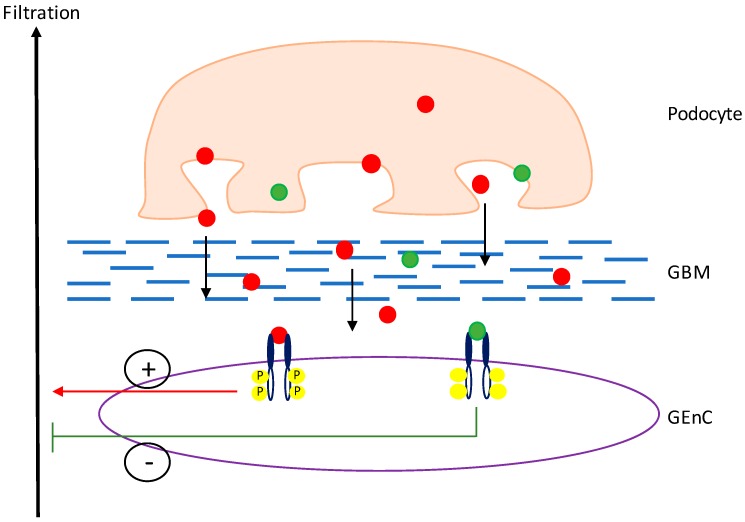
**VEGF-A signalling in the glomerulus.** Both VEGF-A_xxx_ (red dots) and VEGF-A_xxx_b (green dots) are expressed by the podocytes and diffuse through the glomerular basement membrane (GBM) to bind to VEGFR2 on the glomerular endothelial cells (GEnCs), against the flow of filtrate. Upon binding of VEGF-A_xxx_ to VEGFR2, the receptor is phosphorylated, initiating a pro-permeability response. However, upon binding of VEGF-A_xxx_b to VEGFR2, the receptor is not activated and an anti-permeability response is initiated.

Within the glomerulus, VEGF-A is a key regulator of normal function and is predominately expressed in high levels by mature podocytes, which cross the glomerular basement membrane (GBM) to signal to VEGFR-2 on the glomerular endothelial cells (GEnCs) (Figure 3) [30]. In the physiologically normal kidney, despite high levels of podocyte VEGF-A expression, there is no angiogenesis. It is thought that the reason for this is that the podocytes express a balance of the pro- and anti-angiogenic isoforms of VEGF-A to maintain normal functioning of the glomerular filtration barrier (GFB) [21]. In culture, podocytes have been shown to express both VEGF-A_165_ and VEGF-A_165_b mRNA and protein when in a differentiated state [22]. Both isoforms have also been shown to be expressed at the mRNA level in the human kidney cortex [9].

Alterations in glomerular VEGF-A expression is implicated is almost every type of kidney disease; a report by Martini et al. [12] highlighted the strong correlation between VEGF-A expression in the kidney cortex and the estimated glomerular filtration rate (eGFR) in patients with a range of chronic kidney diseases (CKDs). However, it is only recently that the focus has shifted to assess the expression of the VEGF-A_xxx_/VEGF-A_xxx_b splice variant ratio in CKD. There is evidence to suggest that diabetic nephropathy (DN) is associated with a switch in VEGF-A splice isoform expression. Glomerular samples from DN patients have shown an increase in the expression of VEGF-A_xxx_ mRNA relative to VEGF-A_xxx_b mRNA during the later stages of DN, when the kidney function has declined; however, during the early stages of DN, when the kidney is functioning relatively normally, there is an increase in the VEGF-A_xxx_b/VEGF-A_xxx_ ratio [9]. It is suggested that during the early stages of DN, VEGF-A_xxx_b is acting to protect the GFB, preventing a decline in the eGFR.

Another example of the dysregulation of VEGF-A exon 8 splicing in CKD is in Denys–Drash syndrome, where a mutation occurs in the Wilm’s tumour suppression gene, *WT1* [31]. In healthy podocytes, *WT1* binds to the SRPK1 promoter and represses the expression of SRPK1; however, in *WT1* mutant podocytes, a reduction in VEGF-A_165_b is observed due to the mutated *WT1* not transcriptionally repressing SRPK1 [26]. This dysregulation of the VEGF-A splicing balance plays a role in the development of CKD in these patients.

There are several types of ischaemic kidney disease where low VEGF-A expression levels have been shown to contribute to the pathology, which can be rescued by VEGF administration, such as in acute kidney injury [32] and renovascular disease [33]. However, there is no reported evidence of the VEGF-A_xxx_/VEGF-A_xxx_b splicing balance/expression levels in these diseases.

## 4. Manipulation of VEGF-A Splicing as a Potential Therapeutic Avenue in Kidney Disease

**Figure 4:**
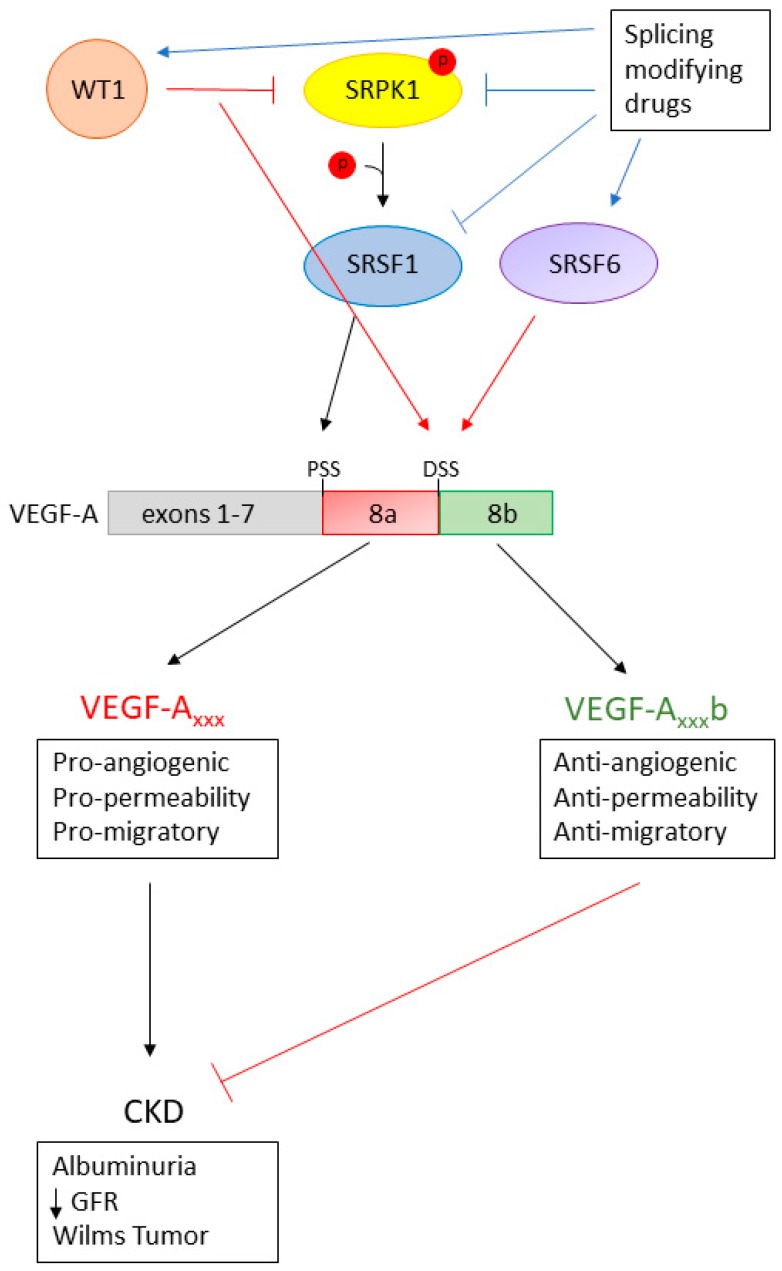
**Switching VEGF-A splicing with splicing-modifying drugs for therapeutic benefit.** SRPK1 is a key regulator of VEGF-A exon 8 splicing; therefore, it is an ideal target for therapeutic modulation of VEGF-A splicing to increase the VEGF-A_xxx_b isoform expression. Splicing-modifying drugs designed to up-regulate *WT1* (thus initiating SRPK1 transcriptional repression), inhibit SRPK1 activation or increase the activation/expression of SRSF6, resulting in a splicing switch to increase the VEGF-A_xxx_b/VEGF-A_xxx_ ratio. As such, they provide a novel therapeutic avenue in the treatment/prevention of chronic kidney disease. CKD: chronic kidney disease; GFR: glomerular filtration rate.

As the number of disease entities known to be associated with AS increases, we are beginning to explore whether manipulation of AS could be used for therapeutic benefit. In terms of VEGF-A splicing in the kidney, the general aim is to try to switch the splicing ratio to increase the anti-angiogenic VEGF-A_xxx_b isoforms, and decrease the pro-angiogenic VEGF-A_xxx_ isoforms (summarised in Figure 4). There has been promising evidence from both in vitro assays and in vivo models to suggest that VEGF-A_165_b is therapeutic in CKD.

In mice, podocyte-specific over-expression of the pro-angiogenic VEGF-A_164_ has been reported to be detrimental to kidney function. Constitutive over-expression of VEGF-A_164_ results in albuminuria at birth and a lack in formation of a fully functional GFB, consistent with the phenotype of congenital nephrosis [34,35]. Similarly, inducible over-expression of VEGF-A_164_ in adult mice results in glomerular disease characterised by proteinuria, increased glomerular water permeability, and GFB abnormalities [36,37], although the severity of the disease phenotype remains inconsistent between different models. When inducible podocyte-specific VEGF-A_164_ over-expressing mice also over-expressed VEGF-A_165_b in a constitutive manner, the functional phenotype was rescued, indicating that the balance of VEGF-A isoforms plays a critical role in the regulation of glomerular permeability [37]. This was also true with the dual insult of inducible podocyte VEGF-A_164_ over-expression and Streptozotocin (STZ)-induced DN; constitutive over-expression of VEGF-A_165_b prevented the onset of albuminuria in this model [9].

On the other hand, low renal expression of VEGF-A has been shown to contribute to disease pathologies, which can be rescued by VEGF-A administration [32,33]. Although the VEGF-A isoform expression levels have not been characterised in specific disease types, a mouse model with podocyte-specific knockdown of VEGF-A has been reported to be detrimental to kidney function. Heterozygous constitutive knockdown of podocyte VEGF-A resulted in nephrotic syndrome and end-stage renal disease (ESRD) by 9 weeks of age, whereas homozygous knockdown resulted in perinatal death [35]. Knockdown of VEGF-A expression in the mature glomerulus has been reported to result in a range of phenotypes, ranging from profound thrombotic glomerular injury [38], to mild albuminuria and ultra-structural changes to the GFB [10]. These differences are likely to result from differing genetic backgrounds and degrees of VEGF-A knockdown. When inducible podocyte-specific VEGF-A knockdown mice over-expressed the VEGF-A_165_b isoform in the podocytes, the kidney injury phenotype was rescued [10]. Our group has recently shown in a quadruple transgenic mouse model that podocyte-specific overexpression of VEGF-A_165_b only, while all other VEGF isoforms are deleted, rescues albuminuria and the increase in water permeability [10].

As described above, DN is associated with dysregulation of VEGF-A splicing. When type I and type II diabetic mice were treated with intraperitoneal injections of VEGF-A_165_b recombinant protein, they did not develop the DN phenotype observed in the diabetic controls [9]. Therefore, VEGF-A_165_b appears to play a therapeutic role in mouse models of CKD, indicating that manipulation of VEGF-A splice isoforms could be a novel therapeutic avenue in CKD. It is important to note that podocyte-specific VEGF-A_165_b over-expression alone resulted in no detrimental changes to kidney function in the long term [39].

A key study examined how the splicing of VEGF-A can be manipulated in cultured *WT1* mutant podocytes. As described above, *WT1* mutant podocytes are unable to transcriptionally repress SRPK1 expression, resulting in increased activation of SRSF1 and high expression of the pro-angiogenic VEGF-A_165_ with reduced expression of the anti-angiogenic VEGF-A_165_b. This alteration in VEGF-A splicing could be reversed by wild-type *WT1*, a knockdown of SRSF1 or SRPK1, and inhibition of SRPK1, thus increasing the expression of the anti-angiogenic VEGF-A_165_b isoform [26].

Podocyte expressed VEGF-A signals to VEGFR-2 located on the GEnCs. Mechanistically, mice over-expressing podocyte-specific VEGF-A_165_b have been shown to have an increase in glomerular VEGFR-2 expression [9]. This is also true for cultured GEnCs treated with VEGF-A_165_b; however, unlike VEGF-A_165_, VEGF-A_165_b is unable to fully phosphorylate VEGFR2 at the tyrosine residues required for full activation, resulting in an inhibition in some of the downstream signalling pathways involved in migration [10]. Interestingly, VEGF-A_165_b can activate pro-survival factors downstream of VEGFR2, Akt and ERK1/2 [10], suggesting that VEGF-A_165_b may either weakly phosphorylate some tyrosine residues of VEGFR2 in a transient manner, or could be acting through other receptors such as VEGFR1. Further research is required to gain more information on the mechanistic action of VEGF-A_165_b in the kidney.

## 5. Discussion

The balance of the VEGF-A_xxx_/VEGF-A_xxx_b appears to be essential in the normal functioning of the GFB. This ratio of splice isoforms has been found to be altered in certain types of CKD, including DN and Denys–Drash syndrome [9,31]. However, manipulation of the pathological balance of isoforms to increase levels of VEGF-A_165_b has been shown to have therapeutic benefit via inhibition of increased angiogenesis and permeability, and through the activation of pro-survival factors [9,10]. This evidence highlights the importance for further investigation into the novel area of the contribution of VEGF-A splicing towards CKD pathogenesis and how we can manipulate the isoform balance for therapeutic benefit. We envision that future studies will provide more insights into the mechanistic aspects of VEGF-A splicing dysregulation in CKD, allowing for the development of a novel class of splicing-modifying therapeutic drugs.
